# Transient alterations in plasma sodium concentrations with NER1006 bowel preparation: an analysis of three phase III, randomized clinical trials

**DOI:** 10.1186/s12876-022-02484-7

**Published:** 2022-09-05

**Authors:** Brooks D. Cash, Christopher Allen, David M. Poppers

**Affiliations:** 1 Division of Gastroenterology, Hepatology, and Nutrition, University of Texas Health Science Center at Houston, 6431 Fannin Street, MSB 4.234, Houston, TX 77030 USA; 2 Salix Pharmaceuticals, 400 Somerset Corporate Blvd., Bridgewater, NJ 08807 USA; 3 New York University Langone Health, 550 First Ave, New York, NY 10016 USA

**Keywords:** Colonoscopy, NER1006, Polyethylene glycol, Plenvu, Sodium

## Abstract

**Background:**

This analysis characterized changes in sodium levels in patients receiving the 1 L polyethylene glycol-based preparation NER1006.

**Methods:**

Data were pooled from three phase III, randomized clinical trials. A post hoc subanalysis included adults who received a 2-day split-dose (evening/morning) NER1006 regimen, a 1-day split-dose (morning only) regimen, or evening-before regimen and had an increase in sodium concentrations from normal to above the upper limit of normal (143–148 mmol/L) at ≥ 1 of three post-treatment visits. Blood samples were collected at baseline, day of colonoscopy (visit 2), 2 ± 1 days post-colonoscopy (visit 3), and 7 ± 1 days post-colonoscopy (visit 4).

**Results:**

A total of 214 of 1028 patients were included. Of the 214 patients, sodium concentration increased from a mean baseline value of 141.8 mmol/L to a mean of 147.1 mmol/L (median increase from baseline of approximately 5 mmol/L). The mean sodium concentration was within normal range at visit 3 (142.3 mmol/L) and visit 4 (142.4 mmol/L), as was the median sodium concentration. Overall, ~ 90% of patients had a normal serum concentration at visits 3 and 4. Based on day of colonoscopy test results, there were four adverse events involving hypernatremia (0.4% of 1028), which were mild and did not require medical intervention; sodium levels returned to normal range by visit 3.

**Conclusion:**

NER1006 was associated with small, transient increases in sodium levels that were not considered clinically significant.

*Trial registration* NOCT (ClinicalTrials.gov: NCT02254486 [registered October 2, 2014]), MORA (ClinTrials.gov: NCT02273167 [registered October 23, 2014]; EudraCT number: 2014-002185-78 [registered August 13, 2014]), DAYB (ClinicalTrials.gov: NCT02273141 [registered October 23, 2014]; EudraCT Number: 2014-002186-30 [registered August 12, 2014])

**Supplementary Information:**

The online version contains supplementary material available at 10.1186/s12876-022-02484-7.

## Background

Osmotically balanced bowel preparations (e.g., polyethylene glycol [PEG] based) were developed to help maintain electrolyte homeostasis during bowel cleansing [1, 2]. The first low-volume (1 L) PEG-based bowel preparation, NER1006 (Plenvu/Pleinvue, Salix Pharmaceuticals/Norgine Ltd), was approved in a European country in 2017 and in the United States in 2018. The efficacy, safety, and tolerability of NER1006 have been reported in three phase III, randomized clinical trials: MORA (Morning Arm), NOCT (Nocturnal Pause Arm), and DAYB (Day Before Arm) [3–5]. In these clinical trials, NER1006 overall cleansing efficacy, evaluated by treatment-blinded central readers, was at least as effective as (i.e., noninferior) standard comparators (2 L PEG plus ascorbate, oral sulfate solution, or sodium picosulfate plus magnesium citrate) [3–5]. Additional analyses demonstrated that high-quality cleansing was superior with NER1006 (2-days evening/morning split dosing [N2D] or 1-day morning only dosing) versus 2 L PEG plus ascorbate (68.0% or 64.0% vs. 50.7%, respectively; *P* < 0.001 for both comparisons) and was greater with NER1006 N2D versus oral sulfate solution (69.9% vs. 63.9%, respectively; *P* = 0.07) [6]. Although NER1006 was found to be safe and well tolerated, sodium levels were elevated following treatment with NER1006, albeit transiently [3, 4, 7]. The current analysis was conducted to further characterize elevations in plasma sodium levels in patients receiving NER1006 in the three phase III trials.

## Methods

The patient population, trial design, and NER1006 dosing regimens for the NOCT (ClinicalTrials.gov: NCT02254486), MORA (ClinTrials.gov: NCT02273167; EudraCT number: 2014-002185-78), and DAYB (ClinicalTrials.gov: NCT02273141; EudraCT Number: 2014-002186-30) trials have been previously published [3–5]. Furthermore, CONSORT information for the three trials was previously documented in those publications and is not repeated herein. Briefly, patients included in the three trials were males or females 18 to 85 years of age undergoing a screening, surveillance, or diagnostic colonoscopy. NER1006 was administered as a 2-day evening/morning split-dosing regimen (NOCT and MORA trials), a 1-day (split-dose) morning-only regimen (MORA trial), or a day-before split-dosing regimen (DAYB trial). In all trials, blood samples were collected at baseline, day of colonoscopy (visit 2), 2 ± 1 days post-colonoscopy (visit 3), and 7 ± 1 days post-colonoscopy (visit 4). This pooled analysis included all patients in the safety population (all patients randomly assigned to treatment for whom patient diary data could not rule out that they received at least one dose of NER1006) who had a normal sodium concentration at baseline and an increase from baseline to above the upper limit of normal (ULN) for sodium (143–148 mmol/L) during at least one of the three post-baseline visits (visits 2–4). Plasma sodium levels were measured via standardized procedures at a local laboratory. Data were analyzed using descriptive statistics.

## Results

**Fig. 1:**
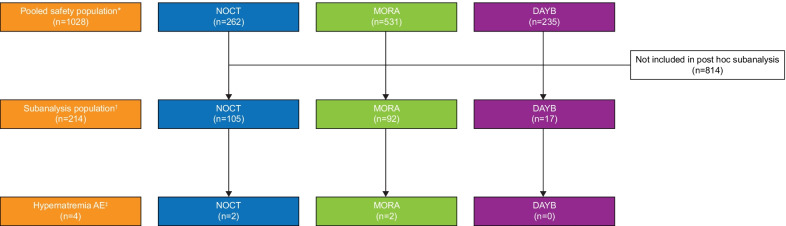
Patient populations. ^*^All patients randomly assigned to treatment for whom patient diary data could not rule out that they received at least one dose of NER1006. ^†^Patients included in the safety population who had a normal sodium concentration at baseline and an increase from baseline to above the upper limit of normal (ULN) for sodium (143–148 mmol/L). ^‡^Reported as an adverse event based on test results on the day of colonoscopy; medical intervention was not required, and sodium levels returned to normal range within 2 ± 1 days post-colonoscopy. *DAYB* Day Before Arm *MORA* Morning Arm *NOCT* Nocturnal Pause Arm

**Fig. 2:**
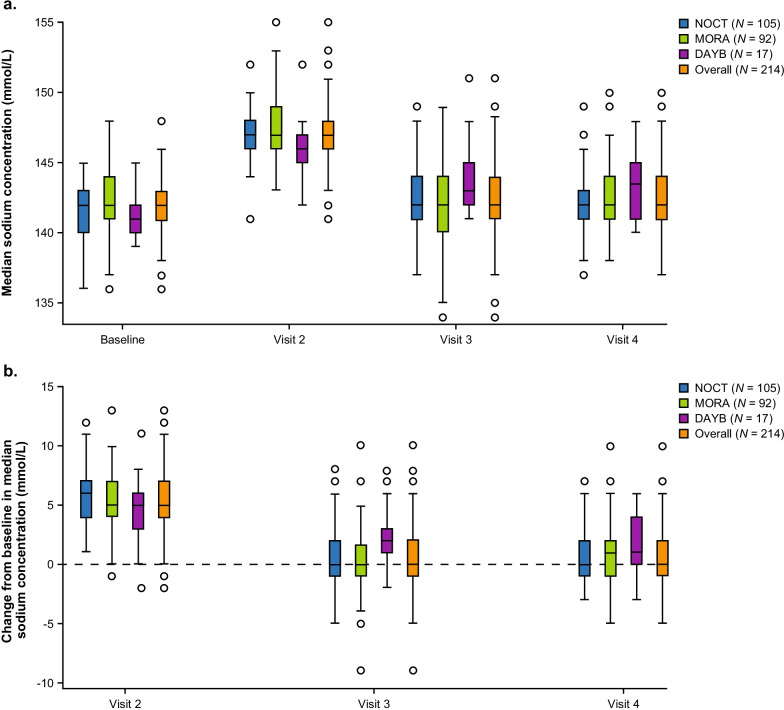
Box and whisker plots of **a** median and **b** change from baseline in median sodium concentrations. Values were determined at baseline, day of colonoscopy (visit 2), 2 ± 1 days post-colonoscopy (visit 3), and 7 ± 1 days post-colonoscopy (visit 4). Box shows median, and upper and lower quartile values; whisker designates upper and lower values. Circles in graph represent outliers. *DAYB* Day Before Arm *MORA* Morning Arm *NOCT* Nocturnal Pause Arm

Of the 1028 patients in the pooled safety population, 214 (20.8%; NOCT, *n* = 105; MORA, *n* = 92; DAYB, *n* = 17) had a normal sodium baseline value and an increase above the ULN for sodium during at least one of the post-baseline visits and were included in the analysis (Fig. 1). Overall, and when grouped by trial, the median sodium concentration increased from normal at baseline to above ULN at visit 2, the day of colonoscopy (Fig. 2a). The sodium concentration in the overall populations increased from a baseline mean value of 141.8 mmol/L to 147.1 mmol/L (Additional file 1: Table S1). The median change (increase) from baseline in sodium levels was approximately 5 mmol/L in the overall population (*n* = 214) and when patients were grouped by trial (Fig. 2b). At visit 2 (day of colonoscopy), 7.9% of patients in the overall subgroup continued to have normal sodium concentrations (Additional file 1: Table S1).

In the overall subgroup, the mean sodium concentration was within normal range (142.3 mmol/L) at visit 3 (2 ± 1 days post-colonoscopy) and remained within the normal range at visit 4 (142.4 mmol/L; 7 ± 1 days post-colonoscopy; Additional file 1: Table S1). Median sodium concentrations returned to the normal range by visit 3 (2 ± 1 days post-colonoscopy) and remained at normal levels at visit 4 (7 ± 1 days post-colonoscopy; Fig. 2). Overall, 89.6% of patients had a normal serum concentration at visit 3 (2 ± 1 days post-colonoscopy) and 89.8% of patients had a normal sodium level at visit 4 (7 ± 1 days post-colonoscopy; Additional file 1: Table S1). For the individual trials, a greater percentage of patients in the NOCT trial (98.1%) experienced elevated sodium concentrations at visit 2 from baseline compared with patients in the MORA (89.1%) and DAYB studies (70.6%; Additional file 1: Table S1). There were four reported adverse events (AEs) involving hypernatremia (0.4% of 1028) during the three trials, all of which were considered of mild intensity (Additional file 1: Table S2). These AEs were considered to be treatment-related, were reported as an AE based on test results on the day of colonoscopy, and did not require medical intervention; sodium levels returned to normal range within 2 ± 1 days post-colonoscopy (visit 3).

## Discussion

The current pooled analysis of three phase III, randomized trials indicated that mean and median sodium electrolyte levels were increased in 214 of 1028 (20.8%) patients on the day of colonoscopy (visit 2) following the use of the NER1006 bowel preparation in a variety of administration regimens [3–5]. However, observed increases in sodium concentration were transient, returning to normal levels within 1 to 3 days post-colonoscopy. The transient increases from baseline in sodium concentrations were small (~ 5 mmol/L) and not considered clinically significant. It is also important to consider baseline sodium levels in order to put these findings into clinical context. In the NOCT trial, more than half of the patients had baseline sodium concentrations above 142 mmol/L, thus requiring only a small increase in sodium concentration to exceed the ULN [8]. However, none of the patients in the NOCT trial with increased sodium levels after NER1006 administration required treatment directed at these modest increases in sodium levels [4]. In all three trials, increased sodium concentrations in the overall populations were not considered to be clinically meaningful [3–5].

The minor, transient changes in sodium levels that may occur in some patients treated with NER1006 are considered to be related to the higher quantity of sodium in the second dose versus the first dose of preparation (i.e., 297.6 mmol [3.2 g] vs. 160.9 mmol [2 g]) and not related to patient volume status or dehydration [8, 9]. Of importance to note, elderly patients may be more susceptible to adverse reactions due to electrolyte imbalances [9]. Also, patients with renal impairment or who are receiving concomitant therapies that affect renal function should be advised on the importance of adequate hydration before, during, and after administration of NER1006, and testing for electrolytes may be considered in this population [9]. Although sodium levels increased at ≥ 1 post-baseline visit in 214 patients with normal baseline sodium levels across the three trials, AEs involving hypernatremia were uncommon (0.4%).

## Conclusion

Increased sodium concentrations from baseline occurred in a subset of adults treated with NER1006 as a bowel preparation for colonoscopy and were transient in nature. As with any bowel preparation, strict adherence to instructions for use is recommended, and additional fluid consumption as prescribed is encouraged to minimize potential adverse effects.

## Supplementary Information

**Additional file 1: Table S1** Summary of sodium concentrations for patients with abnormal serum sodium levels. **Table S2** Clinical profile of patients with adverse event of hypernatremia.

## Data Availability

Qualified researchers interested in obtaining access to trial data should submit a detailed research proposal and data access request to datasharing@bauschhealth.com. For more information, please see https://www.bauschhealth.com/responsibility/access-to-clinical-study-data.

## Abbreviations

AEs: Adverse events
DAYB: Day Before Arm
MORA: Morning Arm
N2D: 2-Days evening/morning split dosing
NOCT: Nocturnal Pause Arm
PEG: Polyethylene glycol
ULN: Upper limit of normal
