# Supplementary material for: Transient alterations in plasma sodium concentrations with NER1006 bowel preparation: an analysis of three phase III, randomized clinical trials
Source: BMC Gastroenterol. 2022 Sep 5;22:412. doi: 10.1186/s12876-022-02484-7 (PMC9446798; doi:10.1186/s12876-022-02484-7)
Supplement: Supplementary file 1 — Additional file 1: Table S1 Summary of sodium concentrations for patients with abnormal serum sodium levels. Table S2 Clinical profile of patients with adverse event of hypernatremia. [file 12876_2022_2484_MOESM1_ESM.docx]

**SUPPLEMENTARY INFORMATION**

**Transient alterations in plasma sodium concentrations with NER1006 bowel preparation: an analysis of three phase III, randomized clinical trials**

Brooks D. Cash^1^, Christopher Allen^2^, and David M. Poppers^3^

^1^University of Texas Health Science Center at Houston, 6431 Fannin Street, MSB 4.234, Houston, TX 77030; ^2^Salix Pharmaceuticals, 400 Somerset Corporate Blvd., Bridgewater, NJ 08807; ^3^New York University Langone Health, 550 First Ave, New York, NY 10016

**Corresponding author email:** [brooks.d.cash@uth.tmc.edu](mailto:brooks.d.cash@uth.tmc.edu)

**Journal:** *BMC Gastroenterology*

**Table S1** Summary of sodium concentrations for patients with abnormal serum sodium levels

| **Trial** | **Visit** | **Patients with normal serum levels, *n*/*n* (%)** | **Mean, mmol/L (SD)** | **Range, mmol/L** |
| --- | --- | --- | --- | --- |
| Overall (*n* = 214) | Baseline | 214/214 (100) | 141.8 (2.0) | 136–148 |
|  | Visit 2 | 17/214 (7.9) | 147.1 (1.9) | 141–155 |
|  | Visit 3^*^ | 189/211 (89.6) | 142.3 (2.6) | 134–151 |
|  | Visit 4^†^ | 150/167 (89.8) | 142.4 (2.4) | 137–150 |
| NOCT (*n* = 105) | Baseline | 105/105 (100) | 141.5 (1.8) | 136–145 |
|  | Visit 2 | 2/105 (1.9) | 146.9 (1.6) | 141–152 |
|  | Visit 3^‡^ | 95/104 (91.3) | 142.2 (2.2) | 137–149 |
|  | Visit 4^§^ | 74/80 (92.5) | 142.1 (2.1) | 137–149 |
| MORA (*n* = 92) | Baseline | 92/92 (100) | 142.2 (2.1) | 136–148 |
|  | Visit 2 | 10/92 (10.9) | 147.6 (2.1) | 143–155 |
|  | Visit 3^¶^ | 81/90 (90.0) | 142.2 (2.8) | 134–149 |
|  | Visit 4^#^ | 68/77 (88.3) | 142.6 (2.6) | 138–150 |
| DAYB (*n* = 17) | Baseline | 17/17 (100) | 141.5 (1.9) | 139–145 |
|  | Visit 2 | 5/17 (29.4) | 146.0 (2.3) | 142–152 |
|  | Visit 3 | 13/17 (76.5) | 143.9 (2.7) | 141–151 |
|  | Visit 4^**^ | 8/10 (80.0) | 143.3 (2.6) | 140–148 |

*SD* standard deviation.

^*^*n* = 211.

†*n* = 167.

^‡^*n* = 104.

^§^*n* = 80.

^¶^*n* = 90.

^#^*n* = 77.

^**^*n* = 10.

**Table S2** Clinical profile of patients with adverse event of hypernatremia

| **Case^*^** | **Intensity of hypernatremia** | **Bowel preparation** | **Recorded sodium level**^†^ **(timing)** | **Additional information** |
| --- | --- | --- | --- | --- |
| 1 | Mild | pm/am NER1006 | 152 mmol/L (day of colonoscopy) | - Sodium level high at screening (145 mmol/L) - Did not require medical intervention - Sodium level normal at both post-colonoscopy visits |
| 2 | Mild | pm/am NER1006 | 149 mmol/L (day of colonoscopy) | - Did not require medical intervention - Sodium level normal at both post-colonoscopy visits |
| 3 | Mild | pm/am NER1006 | 148 mmol/L (day of colonoscopy) | - Did not require medical intervention - Sodium level normal at both post-colonoscopy visits |
| 4 | Mild | am/am NER1006 | 148 mmol/L (day of colonoscopy) | - Did not require medical intervention - Sodium level normal at both post-colonoscopy visits |

^*^All were female with an age range of 46 to 62 years.

^†^Upper limit of normal, 143–148 mmol/L.
